# Toxics or Lures? Biological and Behavioral Effects of Plant Essential Oils on Tephritidae Fruit Flies

**DOI:** 10.3390/molecules26195898

**Published:** 2021-09-29

**Authors:** Valeria Zeni, Giovanni Benelli, Orlando Campolo, Giulia Giunti, Vincenzo Palmeri, Filippo Maggi, Roberto Rizzo, Gabriella Lo Verde, Andrea Lucchi, Angelo Canale

**Affiliations:** 1 Department of Agriculture, Food and Environment, University of Pisa, Via del Borghetto 80, 56124 Pisa, Italy; valeria.zeni@phd.unipi.it (V.Z.); andrea.lucchi@unipi.it (A.L.); angelo.canale@unipi.it (A.C.); 2 Department of Agriculture, University “Mediterranea” of Reggio Calabria, Loc. Feo Di Vito, 89122 Reggio Calabria, Italy; orlando.campolo@unirc.it (O.C.); giulia.giunti@unirc.it (G.G.); vpalmeri@unirc.it (V.P.); 3 School of Pharmacy, University of Camerino, Via Sant’Agostino, 62032 Camerino, Italy; filippo.maggi@unicam.it; 4 CREA Research Centre for Plant Protection and Certification, S.S. 113-km 245.500, 90011 Bagheria, Italy; roberto.rizzo@crea.gov.it; 5 Department of Agricultural, Food and Forest Sciences, University of Palermo, Viale delle Scienze, Ed. 5, 90128 Palermo, Italy; gabriella.loverde@unipa.it

**Keywords:** terpenes, phenylpropanoids, true fruit fly, Integrated Pest Management, tephritid, fumigation, ingestion toxicity

## Abstract

The family Tephritidae (Diptera) includes species that are highly invasive and harmful to crops. Due to globalization, international trade, and human displacement, their spread is continuously increasing. Unfortunately, the control of tephritid flies is still closely linked to the use of synthetic insecticides, which are responsible for detrimental effects on the environment and human health. Recently, research is looking for alternative and more eco-friendly tools to be adopted in Integrated Pest Management (IPM) programs. In this regard, essential oils (EOs) and their main compounds represent a promising alternative to chemical insecticides. EOs are made up of phytoconstituents formed from the secondary metabolism of many plants and can act as attractants or toxics, depending on the dose. Because of this unique characteristic, EOs and their main constituents are promising tools that can be used both in Sterile Insect Technique (SIT) programs and in the “lure and kill” technique, exploiting the attractiveness of the product in the former case and its toxicity in the latter. In this article, current knowledge on the biological and behavioral effects of EOs and their main constituents on tephritid fruit flies is reviewed, mainly focusing on species belonging to the *Anastrepha*, *Bactrocera*, *Ceratitis*, and *Zeugodacus* genera. The mechanisms of action of EOs, their real-world applications, and challenges related to their use in IPM are critically discussed.

## 1. Introduction

True fruit flies (Diptera: Tephritidae) represent an enormous threat to agricultural trade worldwide, causing both quantitative and qualitative damages [1]. Adult females lay eggs under the skin of fruits and vegetables, from which larvae hatch and feed on the decaying flesh of the crop. Infested fruits and vegetables quickly become inedible or drop to the ground [2]. *Anastrepha* Schiner, 1868, *Bactrocera* Macquart, 1835, *Ceratitis* Macleay, 1829, *Dacus* Fabricius, 1805, *Rhagoletis* Loew, 1862, and *Zeugodacus* Hendel, 1927 are among the most economically relevant genera [3], attacking a wide array of important fruit and vegetable crops, such as mango, peach, apple, pear, and citrus, just to cite some [1]. Therefore, many fruit-producing countries have imposed quarantine restrictions on the import of products from regions where infestations by particular fruit fly species occur, and/or require that fruits and vegetables undergo postharvest treatments (e.g., fumigation, heat/cold treatment, and irradiation) before their importation is allowed [2]. The main goal of control programs is to eradicate or suppress these pests. Control tools involved in these programs include insecticides of synthetic (e.g., organophosphates), and natural origin [4,5], as well as biotechnical tools (e.g., Sterile Insect Technique (SIT) and Male Annihilation Technique (MAT)) [6,7,8], and biological control [9,10,11]. Nowadays, the control of true fruit flies mostly relies on synthetic insecticides, whose toxicity and non-biodegradable properties have led to the development of resistant strains and/or species [12,13], ecological imbalances, and toxicological hazards for humans [14]. Since crop protection is moving from an agrochemical curative approach to a more balanced and sustainable one, the research community aims to find new techniques to reduce the detrimental effects of pesticides [15]. In this framework, bioactive compounds of natural origin represent an extraordinary source of molecules with proven efficacy against organisms harmful to crops [14,16,17]. In particular, plant essential oils (EOs), and their main bioactive constituents, can exert their activity on a large number of insect species, through multiple modes of action [18,19,20].

**Figure 1:**
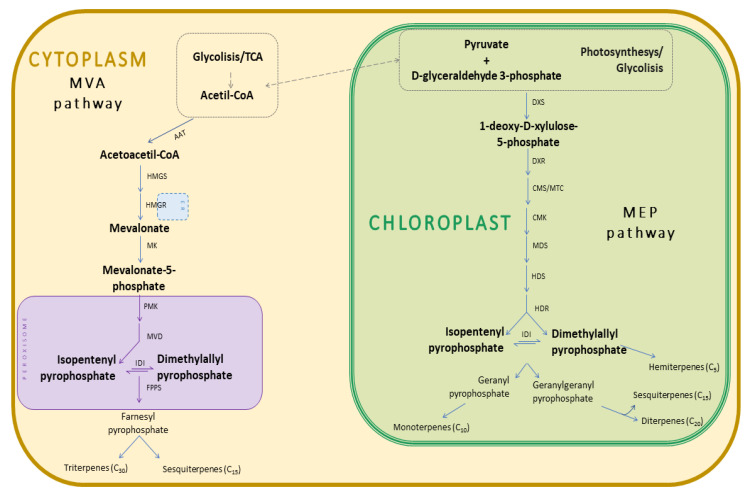
Terpenes and terpenoids are the most common constituents of EOs. The biosynthesis of these compounds in plants occurs via two main pathways: the mevalonate (MVA) pathway in the cytosol and the methylerythritol phosphate (MEP) pathway in the plastids. Both pathways produce the C_5_ precursors isopentenyl pyrophosphate (IPP) and dimethylallyl pyrophosphate (DMAPP), which are condensed via geranyl/farnesyl pyrophosphate synthase to give the C_10_ (geranyl diphosphate, GPP), C_15_ (farnesyl diphosphate, FPP), and C_20_ (geranylgeranyl pyrophosphate) precursors for isoprenoid production. Depending on the number of carbons, isoprenoids are grouped in monoterpenes (C_10_), sesquiterpenes (C_15_), diterpenes (C_20_), triterpenes (C_30_), etc., with the first two groups being the main constituents of EOs. Monoterpenes and diterpenes tend to be formed in the plastids, where unique cyclases produce the ring structures. Aromatic compounds, including phenylpropanoids, are less common and are derived mainly from the shikimate pathway, but a few phenols, such as carvacrol and cuminaldehyde, are derived from terpene biosynthesis by desaturation. The graphical representation of MVA and MEP pathways are adapted and re-drawn from Vickers et al. [27]. MVA pathway enzymes abbreviations: **AAT**, acetyl-CoA C-acetyltransferase; **HMGS**, hydroxymethylglutaryl-CoA synthase; **HMGR**, hydroxymethylglutaryl-CoA reductase; **MK**, mevalonate kinase; **PMK**, phosphomevalonate kinase; **MVD**, diphosphomevalonate decarboxylase; **IDI**, isopentenyl diphosphate isomerase; **FPPS**, farnesyl pyrophosphate synthase. MEP pathway enzymes abbreviations: **DXS**, deoxyxylulosephosphate synthase; **DXR**, deoxyxylulosephosphate reductoisomerase; **CMS**, diphosphocytidylmethylerythriol synthase; **CMK**, diphosphocytidylmethylerythriol kinase; **MDS**, methylerythriol cyclodiphosphate synthase; **HDS**, hydroxymethylbutenyl diphosphate synthase; **HDR** hydroxymethylbutenyl diphosphate reductase; **IDI**, isopentenyl diphosphate isomerase.

EOs are made up of phyto-constituents formed from the plant secondary metabolism. They can be obtained from a plant raw material by steam distillation and hydrodistillation of the cold pressing, as in the case of citrus. The heating producing the vapor during distillation can be obtained by conventional heating, with a temperature gradient from outside to inside, or by microwave heating, with a gradient from inside to outside [21]. Usually synthetized and produced by specialized secretory tissues (e.g., glandular trichomes, channels, pockets, and idioblasts) and present in all plant organs, they play a pivotal ecological role in plants [22]. They can attract seed disseminators and pollinators, repel predators, inhibit germination, provide plant to plant signaling, and are also responsible for plant thermotolerance and ozone quenching [20,23,24]. From a biochemical point of view, EOs are a mixture of volatile molecules, with monoterpenoids, sesquiterpenoids, and phenylpropanoids being the most representative chemical classes [20,25,26]. Typically, EOs are characterized by two or three main compounds at high concentrations (e.g., mostly ranging from 20 to 85%) and other molecules at trace levels [20]. Both play an important role in determining the EO biological activities [25]. Their synthesis occurs in plastids or cytoplasm of plant cells following two biochemical pathways, i.e., the mevalonic acid pathway (MVA) and the methyl-D-erythritol-4-phosphate pathway (MEP) [20] (Figure 1).

The mevalonic acid pathway occurs in the cytoplasm and uses acetyl-CoA as a precursor; its products are sesquiterpenes (C_15_), triterpenes (C_30_), and polyterpenes. MEP pathway operates in the plastid and uses pyruvate and glyceraldeyde-3-phosphate (3-PGA) as precursors, leading to the formation of monoterpenes (C_10_), diterpenes (C_20_), and tetraterpenes (C_40_). Based on their vapor pressure, monoterpenes and sesquiterpenes are common constituents of EOs while the other groups are not volatile apart from some diterpenes. In both cases, the terpene skeleton originates from the head-to-tail condensation of isoprene (2-methylbuta-1,3-diene)(C_5_H_8_) units [28], while in some cases the addition of functional groups, mostly containing oxygen, gives rise to the so-called terpenoids. In the case of phenylpropanoids, the shikimate pathway is used from the cell to synthesize in the cytoplasm C_6_C_3_ units such as cinnamic acid and its derivatives such as chavicol, eugenol, anethole, and myristicin.

The biological activity of EOs strictly depends on their chemical composition, which varies according to the plant parts used, extraction technique, drying method, plant phenological stage, harvesting season, plant age, soil composition, and environmental conditions in which the plant grows [29]. The biological activity of EOs is expressed in various ways ranging from toxic to repellent effects, to modification of pest behavior and/or physiology [30]. Depending on the dose, an EO may act as an attractant, a repellent, or a toxin [31]. The dualistic property of EOs has been poorly documented, but overall, the transition from attractiveness to repellence and then to toxicity is a function of the treated species, the composition of the EO, and, also, the concentration. As a general scheme, EOs are toxic at the highest concentration, and by decreasing their concentration they become repellent or attractive to insects. In addition, EOs are also characterized by a neutral phase in which there is a balance between the attractant and repellent effect [31]. Furthermore, this dualistic property is also a characteristic of individual molecules contained in EOs. For instance, both limonene and *p*-cymene, isolated from the fruit of *Mangifera indica* L. (Anacardiaceae), act as strong attractants towards adults of the Mediterranean fruit fly, *Ceratitis capitata* (Wiedemann) (medfly) [32], but toxicity assays revealed that EOs rich in limonene are toxic towards adults of this species [33].

Some EOs show an attractive power towards insects, being exploited by the latter as aggregation signals. Several studies investigated the role of plant volatiles in male aggregation behavior, with a particular reference to the site selection for leks [34,35,36]. As reported by Segura et al. [36], secondary plant metabolites may affect the sexual behavior and communication of Tephritidae, enhancing male signaling behavior, the attraction of females, and their chances to mate.

Historically, EOs have been widely used in herbal and traditional medicines, as well as in culinary traditional dishes [37]. However, their promising biochemical properties against arthropod pests and the simultaneous limited or absent toxicity to mammals, including humans, encouraged the research about their use in biological control programs as an environmental-friendly alternative to conventional pesticides (Box 1).

## 2. Biological Activity of Essential Oils and Their Constituents

EOs are a natural source of active compounds with antifungal, anti-mycotoxigenic, insecticidal, and herbicidal potential [38]. In particular, EOs show various biological properties including attractive, antifeedant, deterrent, and toxic effects on numerous phytophagous insects [25,39]. Toxic and repellent activity of EOs were tested on several pests of agricultural and medical interest [40,41,42,43,44], including tephritid flies [5,45,46,47,48,49]. In the present review, we summarized the current status of the experiments carried out on Tephritidae, in which the toxic and attractive activities of EOs and their main constituents were evaluated. 

### 2.1. Essential Oils as Lures

Plants are used as “*rendezvous*” sites in true fruit flies mating and plant volatiles play a key role in male aggregation behavior and site selection for leks [34,35,50]. Indeed, secondary plant metabolites, including EO constituents, can affect the sexual behavior and communication of many insects, such as Tephritidae [36] (Box 2).

#### 2.1.1. α-Copaene: A Booster of Mating Success in *Ceratitis* spp. and *Anastrepha* spp.

One of the most studied compounds for its attractiveness on Tephritidae is α-copaene, a tricyclic sesquiterpene commonly found in several host plants of *C. capitata,* such as sweet orange (*Citrus sinensis* L.) (Rutaceae), guava (*Psidium guajava* L.) (Myrtaceae), papaya (*Carica papaya* L.) (Caricaceae), and mango (*Mangifera indica* L.) (Anacardiaceae). Commonly released by several plants as a secondary metabolite, α-copaene facilitates the location and subsequent formation of leks for males, while it works as an attractant towards females [34,51,52]. Since this compound is hard to synthesize and quite expensive, several studies have been conducted on α-copaene-containing EOs (e.g., *Leptospermum scoparium* Forst. and Forst. (Myrtaceae), *Zingiber officinalis* Roscoe (Zingiberaceae), *Angelica archangelica* L. (Apiaceae), and *C. sinensis*) [34,35,52,53]. α-Copaene is a chiral molecule and can occur in two different conformations: (+)-α-copaene, known to be responsible for the male attraction, and (−)-α-copaene, less active but most abundant in nature [54,55].

In *C. capitata* males, the mating success might be enhanced thanks to an increase of sexual signalings, such as pheromone calling. In a study by Nishida et al., [34], males of *C. capitata* displayed pheromone calling more frequently on artificial leaves coated with α-copaene than on non-treated leaves, hypothesizing that α-copaene may act as a cue of lek sites. Similarly, males exposed to ginger rhizome oil (*Z. officinalis*), rich in α-copaene, spent more time in pheromone calling than non-exposed ones [36,56]. In addition, the ginger EO seems to enhance the copulatory success of males, as the exposition to the EO modified their cuticular compounds, changing the aroma of their exoskeleton. Similarly, to perfume, the attractiveness towards females then increased [57]. The mating success of *C. capitata* males seems to be enhanced also after the exposure to grapefruit oil, *Citrus paradisi* Macfad. (Rutaceae), containing α-copaene [58].

Recently, the possibility that α-copaene can synergically interact with other compounds was hypothesized as well. In a study conducted by Niogret et al. [59], the attraction levels of six different EOs (*A. archangelica, Z. officinalis, C. sinensis*, *L. scoparium, Piper cubeba* Vahl (Piperaceae), and *Melaleuca alternifolia* (Maiden & Betche) Cheel (Myrtaceae)) were not directly correlated with their richness in α-copaene, supporting the hypothesis that this molecule does not work alone, but acts synergically with other compounds, such as myrcene, linalool, geraniol, camphene, and α-terpineol [59]. Myrcene, linalool, and geraniol are precursors of *C. capitata* sex pheromone, that may explain their attractiveness on fruit flies [60,61,62]. The lure potential of *Z. officinalis* and *C. sinensis* EOs was also investigated on males of *Ceratitis rosa* Karsch, showing that exposure to both EOs increases the mating success in this species [63]. Interestingly, the orange EO (*C. sinensis*) was efficient only if strictly correlated with a proper diet (sugar + protein), suggesting that the intake of protein may interfere with the attractiveness of EOs and their main compounds [63]. Likewise in *C. capitata*, the calling activity of males exposed to *C. sinensis* was higher if males were fed on a diet based on protein and sugar [36].

The attractiveness of α-copaene towards *Anastrepha* species is controversial. For instance, the mating success of *A. fraterculus* (Wiedemann) males increased after the exposure to citrus and guava aroma, while no differences were noted for males exposed to mango [64,65]. Males exposed to guava odor increased signaling of 35–40%, because of increased release of sex pheromone. However, the female response to cuticle extracts of exposed and unexposed males did not differ significantly, showing that no “perfume effect” occurred after the exposure to guava odor [65]. The mating success of males of *Anastrepha ludens* (Loew) and *Anastrepha serpentina* (Wiedemann) is enhanced after the exposition to grapefruit oil, *C. paradisi,* and ginger EO, respectively, [66,67], while no effect has been observed for ginger EO on the mating success of males of *A. ludens* and *Anastrepha obliqua* (Macquart) [67].

Little is known about the role of α-copaene in flies belonging to the genus *Bactrocera*. Only one study reports that olive drupe with a high amount of (+)-α-copaene favored the oviposition of *B. oleae* (Rossi) females, whereas the increase of (−)-α-copaene did not provide any differences in the host choice [68].

#### 2.1.2. Methyl Eugenol: Rendezvous Cue or Sex Pheromone Precursor?

As for α-copaene in *Ceratitis* spp., methyl eugenol (ME) exerts a similar action towards flies of the genus *Bactrocera*. ME is a phenylpropanoid, found in many plant species, such as *Croton malambo* H. Karst. (Euphorbiaceae), *Cinnamomum cordatum* Kosterm. (Lauraceae), *Piper divaricatum* G. Mey. (Piperaceae), *Pimenta racemosa* (Mill.) J. W. Moore (Myrtaceae), and several species of the genus *Melaleuca* (Myrtaceae) [69,70,71,72,73,74,75,76,77,78].

The attractiveness of phenylpropanoids, in particular ME, towards *Bactrocera* flies is known since the beginning of the 20th century [79]. Several studies considered ME as an essential source for male pheromone production [80,81]. In males of *Bactrocera dorsalis* (Hendel) and *Bactrocera papayae* Drew & Hancock, after ingestion, ME is converted to 2-ally-4,5-dimethoxyphenol (DMP) and (*E*)-coniferyl alcohol (CF) and stored in rectal glands [82,83]. Both compounds are then released during the courtship period at dusk, as a part of the sex pheromone of these species [82,83,84,85]. Indeed, only males of *B. dorsalis* that ingested the ME-containing substances [80] or males exposed to ME produce a sex pheromone containing metabolites of this compound [82,83]. Additionally, *Bactrocera correcta* (Bezzi) and *Bactrocera carambolae* Drew & Hancock convert ME before its stockage into the rectal glands [86,87]. ME plays a prominent role in promoting intersexual communication and enhances the mating performance of several *Bactrocera* species. *B. dorsalis* males exposed to ME displayed a higher wing fanning and a more efficient calling if compared with unexposed males [81]. In addition, the effect of ME seems relatively long-lasting, as treated males kept showing a mating advantage towards unexposed ones 35 days after the ingestion [81]. ME-fed males attracted significantly more males in *B. dorsalis* [81], *B. carambolae* [85], and *Bactrocera umbrosa* (Fabricius) [88] and ME-fed males promoted aggregation behavior in *B. carambolae* and *B. umbrosa* [85,88]. Interestingly, ME-deprived males fed also on anal secretions of ME-fed males, which contains (*E*)-coniferyl alcohol (CF) along with endogenously produced pheromonal compounds [85].

However, in contrast to the other abovementioned species, males of *B. cacuminata* (Hering) do not gain any mating benefits from the ingestion of ME [89], leading to query the role of ME as a pheromone precursor. The biosynthetic pathway of *B. cacuminata* sex pheromone is independent of the ingestion of ME and this compound does not play any role in the male pheromone system of this species [89,90,91]. In this context, fly response to kairomones, such as ME or other phenylpropanoids, may have an ancestral origin, and be linked rather to its function as a *rendezvous* stimulus [51,92].

#### 2.1.3. The Role of Cue Lure and Raspberry Ketone in *Bactrocera* and *Zeugodacus* Species

Some *Bactrocera* species, such as *B. tryoni* (Froggatt), respond to cue lure (CL) and its hydrolysis product, raspberry ketone (RK) which causes a similar response also in *Zeugodacus* species [93]. As for other compounds, males show far more attraction to CL/RK than females [36]. RK occurs in a plethora of different plant species, while CL has always been considered as a synthetic compound until its recent detection in some *Bulbophyllum* orchids [93]. Once ingested, RK accumulates in rectal glands of *Zeugodacus cucurbitae* (Coquillet), *B. caudata* (Fabricius), and *B. tryoni*, while CL is accumulated after its hydrolyzation [94,95]. Different from ME, in *B. tryoni*, RK is incorporated into sex pheromone without modifications [95].

Both CL and RK influence the mating success of *Z. cucurbitae*, though its effect is noticeable only at short intervals of time, the advantage being evanished just 3 days after the exposure [96]. The CL-mediated mating enhancement lasts a little bit longer in *B. tryoni*, conferring to males an advantage of 3 days after feeding [96]. Albeit the contribution of CL/RK to the sex pheromone appears clear, its role in the physiology and behavior of Tephritidae is not fully explained. Overall, CL/RK increases the activity of males, enhances male calling frequency, male locomotor activity, and successful mating, but further studies are needed to shed light on other possible roles played by these compounds [96,97].

#### 2.1.4. Other Compounds: α-Pinene and Zingerone

α-Pinene is another widely distributed monoterpene, attractive to *B. oleae* males. The importance of α-pinene is not only connected to its presence as a major or minor compound in different EOs but as a part of *B. oleae* female sex pheromone [98]. Similar to α-copaene, ME, and CL/RK, α-pinene attracts males, while it is weakly efficient towards females [98,99]. Males exposed to α-pinene mated more frequently and for a longer time [100].

As the name might recall, zingerone (ZG) is the molecule responsible for the pungency of ginger. Unfortunately, its role in the tephritid mating sequence has not been deeply investigated yet. ZG is known to attract both ME- (e.g., *B. dorsalis*, *B. carambolae*, and *B. umbrosa*) and RK/CL- (e.g., *Z. cucurbitae* and *Zeugodacus tau* (Walker)) responsive fruit fly species (see [101]). In *Z. cucurbitae*, males fed with zingerone were more attractive to both females and other males [102], even if no evidence on mating enhancement was detected on males fed with ZG, or on females mated to ZG-fed males (i.e., longevity, fecundity, and egg viability) [103]. On the other hand, males of *B. tryoni* increased their mating success because of ZG ingestion, even if the attractiveness of their sex pheromone remained unaltered [96]. Transcriptomic studies on *B. tryoni* also revealed that ZG-feeding resulted in an up-regulation of genes associated with male aggression, pheromone synthesis, mating, and accessory gland proteins regulation as well as enrichment of several energy metabolic pathways [104]. A possible explanation is that in *B. tryoni* ZG is partially converted into RK, a compound known as a booster of mating, while only a small amount of ZG ingested by *Z. cucurbitae* is converted to zingerol, whose effect on behavioral traits of *Z. cucurbitae* is still unknown. A recent study suggested that ZG could play an important role in the sexual selection of *Z. tau*, also known as pumpkin fruit fly [105]. Males of *Z. tau* fed on ZG significantly increased attraction of conspecific females and ZG also improved males mating success, because of increasing wing fanning, mounting, and sexual signals [105]. In addition, ZG effect was dose-dependent: at low concentration, it acted primarily as a metabolic enhancer of courtship activities. On the other hand, a higher dose of ZG not only increased *Z. tau* male signaling activities but also made their sexual signals more attractive.

### 2.2. Essential Oils as Tephritid Repellents and Oviposition Deterrents

Repellents are chemical substances able to modify the insect behavior and prevent them from flying to, landing/walking on, or ovipositing on a given source [106,107]. Studies about EO repellent properties have been chiefly carried out on mosquitoes [17,108] and stored-product pests [109,110], just to cite two major groups. Few studies investigated the repellent and oviposition-deterrent effects of EOs and their main compounds towards true fruit flies [111,112,113,114,115] (Table 1). On the other hand, many studies have focused their attention on the repellent properties of other botanical extracts, particularly towards *Bactrocera* species [116,117,118,119,120,121,122,123]. 

Among the EOs investigated on tephritids, the repellent properties of citrus peel EO towards *C. capitata* are noteworthy. Several studies pointed out the controversial role of citrus peel EO, which may elicit neutral, deterrent, and stimulating effects on medfly oviposition behavior [124,125,126]. As reported by Ioannou et al. [125], citrus EOs operate in a contradictory sequential mode regarding medfly oviposition decisions. Firstly, they attract females and trigger oviposition, then, once a female attempts to oviposit and the ovipositor touches the peel, the deterrent effect arises. Testing out the components of citrus peel EO, linalool mainly found in unripe fruits, resulted responsible for the deterrent effects [125]. These results are corroborated by Papanastasiou et al. [115], who highlighted that linalool negatively affected the acceptance of female medflies in laboratory bioassays and elicited female repellent activity in the field. Interestingly, the male response to linalool is in complete contrast. Males are attracted to it, and individuals exposed to linalool also gain an advantage during mating [59]. As linalool, also EOs of some plant species in the genus *Tagetes* (Asteraceae) may elicit a controversial action depending on the sex. Indeed, as reported by Lopéz et al. [113], *T. minuta* EO acts as an attractant to males and as a repellent to females.

**Table 1:** Essential oils (EOs) were examined for repellent activity on Tephritidae flies. In addition to tested essential oil, the mode of action and the observed repellent effect are reported.

Targeted Species	Tested EO/ Compound	Botanical Family/ Chemical Class	Observed Effect	References	Notes
***Anastrepha*** ***fraterculus***	*Cymbopogon winterianus*	Poaceae	Oviposition deterrent	[111]	The oviposition deterrent effect was noted only on treated apples
***Bactrocera*** ***cucurbitae***	*Cymbopogon citratus*	Poaceae	Oviposition deterrent Repellent	[127]	-
***Bactrocera*** ***cucurbitae***	*Cymbopogon giganteus*	Poaceae	Oviposition deterrent Repellent	[127]	-
***Bactrocera*** ***cucurbitae***	*Cymbopogon nardus*	Poaceae	Oviposition deterrent Repellent	[127]	-
***Bactrocera*** ***cucurbitae***	*Cymbopogon schoenanthus*	Poaceae	Oviposition deterrent Repellent	[127]	-
***Bactrocera*** ***tryoni***	*Citrus limon*	Rutaceae	Oviposition deterrent Repellent	[112]	Additionally, vegetable oils of *Carthamus tinctorius*, *Gossypium herbaceum*, *Linum usitatissimum*, and *Azadirachta indica* were tested
***Bactrocera*** ***tryoni***	*Corymbia citriodora*	Myrtaceae	Oviposition deterrent Repellent	[112]	Additionally, vegetable oils of *Carthamus tinctorius*, *Gossypium herbaceum*, *Linum usitatissimum*, and *Azadirachta indica* were tested
***Bactrocera*** ***tryoni***	*Eucalyptus staigeriana*	Myrtaceae	Oviposition deterrent Repellent	[112]	Additionally, vegetable oils of *Carthamus tinctorius*, *Gossypium herbaceum*, *Linum usitatissimum*, and *Azadirachta indica* were tested
***Bactrocera*** ***tryoni***	*Eucalyptus radiata*	Myrtaceae	Oviposition deterrent Repellent	[112]	Additionally, vegetable oils of *Carthamus tinctorius*, *Gossypium herbaceum*, *Linum usitatissimum*, and *Azadirachta indica* were tested
***Bactrocera*** ***tryoni***	*Eucalyptus dives*	Myrtaceae	Oviposition deterrent Repellent	[112]	Additionally, vegetable oils of *Carthamus tinctorius*, *Gossypium herbaceum*, *Linum usitatissimum*, and *Azadirachta indica* were tested
***Bactrocera*** ***tryoni***	*Leptospermum petersonii*	Myrtaceae	Oviposition deterrent Repellent	[112]	Additionally, vegetable oils of *Carthamus tinctorius*, *Gossypium herbaceum*, *Linum usitatissimum*, and *Azadirachta indica* were tested
***Bactrocera*** ***tryoni***	*Mentha* × *piperita*	Lamiaceae	Oviposition deterrent Repellent	[112]	Additionally, vegetable oils of *Carthamus tinctorius*, *Gossypium herbaceum*, *Linum usitatissimum*, and *Azadirachta indica* were tested
***Bactrocera*** ***tryoni***	*Melaleuca teretifolia*	Myrtaceae	Oviposition deterrent Repellent	[112]	Additionally, vegetable oils of *Carthamus tinctorius*, *Gossypium herbaceum*, *Linum usitatissimum*, and *Azadirachta indica* were tested
***Ceratitis*** ***capitata***	*Citrus limon* cv. “Lunario”	Rutaceae	Repellent	[114]	Field bioassays
***Ceratitis*** ***capitata***	*Citrus limon* cv. “Interdonato”	Rutaceae	Repellent	[114]	Field bioassays
***Ceratitis*** ***capitata***	*Tagetes minuta*	Asteraceae	Repellent	[113]	The repellent effect was observed towards females of *C. capitata*. Differently, males were attracted by this EO
***Ceratitis*** ***capitata***	Linalool	Terpene	Oviposition deterrent	[115]	One of the major compounds of citrus peel EOs. Females are repelled by this compound while males gain a mating advantage

In no-choice bioassays, *Citrus limon* (L.) Osbeck (Rutaceae) resulted in both repellent and oviposition deterrents also towards *B. tryoni* females [112]. In this study, the repellent properties of eight different EOs (i.e., *C. limon*, *Corymbia citriodora* (Hook.) K.D.Hill & L.A.S.Johnson (Myrtaceae), *Eucalyptus staigeriana* F.Muell. ex F.M.Bailey (Myrtaceae), *E. radiata* Sieber ex DC (Myrtaceae), *E. dives* Schauer (Myrtaceae), *Leptospermum petersonii* F.M.Bailey (Myrtaceae), *Mentha × piperita* (Lamiaceae), and *Melaleuca teretifolia* Endl. (Myrtaceae)) were investigated; at the highest dose (10 mL/L), all the EOs caused a significant reduction in landings of *B. tryoni* females on treated apples, in particular, the *M. × piperita* EO elicited the strongest repellent effect. However, due to their low persistency, all the tested EOs prevented *B. tryoni* attack only for a short period [112].

### 2.3. Essential Oils as Toxins

The insecticidal activity of EOs against crop and stored-product pests, as well as on arthropod vectors has been widely investigated [30,128,129]. Hereafter, we review the results achieved using EO treatments against tephritid flies according to their application method (i.e., fumigation, contact, and ingestion). In detail, we emphasize the most promising results for every genus, highlighting, similarities or divergences between tested pest and/or EO plant species.

The EOs used in toxicity assays have been extracted mainly from aerial parts, such as fresh or dried leaves, followed by seeds, schizocarps, peels, and roots. The common extraction methods are hydro-distillation and steam distillation [45,130,131].

#### 2.3.1. Fumigant Toxicity of Essential Oils and Their Main Compounds

Fumigation bioassays consist of saturating the air in which the insects reside with the toxic compound to be tested; tests are typically carried out in airtight chambers and last from few hours to a day [45,46,48,49].

According to the revised literature, the insecticidal activity of 35 EOs and 13 compounds belonging to terpenes and phenylpropanoids have been investigated through fumigation so far. The most studied plant families were Lamiaceae, Asteraceae, Myrtaceae, Apiaceae, and Rutaceae. Most of the tests were conducted against *C. capitata* (8 EOs and 11 compounds), followed by *Bactrocera* (9 EOs and 3 compounds), *Anastrepha* (5 EOs and 2 compounds), and *Z. cucurbitae* (4 EOs and 4 compounds) (Table 2 and Table 3).

In some cases, EOs tested by fumigation showed a promising result against true fruit flies. For instance, the EO of *Mentha pulegium* L. (Lamiaceae), caused the death of 100% of *B. oleae* adults after 1.5 h of exposure; the EO of *Eucalyptus* spp. L’Hér. (Myrtaceae) elicited a comparable result on adults of *B. dorsalis* and *Z. cucurbitae* [130,132].

Basil EO (*Ocimum basilicum* L., Lamiaceae) is one of the EOs whose toxicity by fumigation has been among the most studied on fruit flies, being tested towards adults of *B. cucurbitae* [45], *B. dorsalis* [45,130], *C. capitata* [45], and *Z. cucurbitae* [130]. The toxicity of basil EO is likely related to its main compounds: linalool, estragole, and *trans*-anethole [45,130]. Linalool has multiple targets, as it may act as GABA_A_R agonist, acetylcholinesterase (AChE) inhibitor, or be involved in cellular oxide-reduction mechanisms [133,134]; estragole has a weak agonistic effect on the GABA_A_R and AChE, while *trans*-anethole acts on AChE [133,135]. Investigating the toxic action of these compounds on *B. cucurbitae*, *B. dorsalis* and *C. capitata*, Chang et al., [45] highlighted that *trans*-anethole and estragole are more toxic than basil oil and linalool, and that the rapid action of these compounds may vary according to fruit fly species. Albeit in lower concentrations, basil EO also contains 1,8-cineole, a monoterpene cyclic ether found abundantly in nature [136]. This compound may be responsible for the toxicity against *C. capitata* of EOs extracted from rosemary *(Rosmarinus officinalis* L., Lamiaceae) and lavender (*Lavandula latifolia* Medik. (*=Lavandula angustifolia* Moench) Lamiaceae) leaves [49] and its toxic activity has been already acknowledged against other pests of agricultural interest [137,138,139,140]. So far, studies on the toxicity by fumigation of EOs and their main compounds on true fruit fly immature stages have been rare and often inconclusive [141,142]. In general, larvae are more susceptible to fumigation than eggs, as the eggshell often acts as an excellent barrier to insecticides, fungal pathogens, and fumigants [143,144]. For instance, the toxicity of the EO of *Ammoides verticillata* (Desf.) Briq. (Apiaceae) towards *B. oleae* differs depending on the developmental stage of the fly. *A. verticillata* EO kills 100% of *B. oleae* adults at a concentration of 2 μL/L air, whereas a higher dosage (12 μL/L air) is necessary to kill the 80% of larvae and pupae [142]. *A. verticillata* is characterized by the presence of limonene and carvacrol, both known to exert an insecticidal activity towards several arthropod pests [145,146]. Though the mode of action is still unknown, according to Khanikor et al. [147], the terpenic compounds of this EO act on the AChE and the octopaminergic systems. An anti-AChE effect has also been hypothesized as the mechanism of action of *M. alternifolia* EO towards *C. capitata* adults [48]. Interestingly, the fumigant toxicity of *M. alternifolia* EO is greater towards *C. capitata* (LC_50_: 2.239 μL oil/L air) than towards its parasitoid, the braconid *Psyttalia concolor* (Szépligeti) (LC_50_: 9.348 μL oil /L air), supporting the safety of this EO to non-target organisms.

Several studies have addressed the toxic action of EOs to their main compounds, monoterpenoids, and phenylpropenes [45,134,141,148]. The fumigant toxic effect has been tested for several monoterpenoids, such as α-pinene, linalool, carvacrol, eugenol, *p*-cymene, cinnamaldehyde, anethole, terpineol, and cuminaldehyde [134]. Among these molecules, linalool, carvacrol, eugenol, and terpineol presented a greater activity on *C. capitata* [134]. Eugenol may interact with octopamine receptors [149,150], whereas thymol, carvacrol, and α-terpineol, can interact with tyramine, a precursor of octopamine [151]. Thymol is also able to bind to GABA receptors associated with chloride channels located on the membrane of postsynaptic neurons and disrupts the functioning of GABA synapses [152]. The monoterpene (*R*)-carvone proved to be an efficient toxicant towards second-instar larvae of *B. zonata*, together with (*R*)-camphor and (1*R*,2*S*,5*R*)-menthol [148]. These compounds cause adult malformation and decrease female longevity, affecting the hormonal balance of the adult fruit flies. These monoterpenes may act similarly to growth-regulating hormones, such as juvenile hormones [153,154]. In addition, (*R*)-camphor, (*R*)-carvone, and (1*R*,2*S*,5*R*)-menthol show destructive effects on ovary and ovarioles of *B. zonata* females, where they can lead to complete inhibition of oviposition [148].

**Table 2:** Essential oils (EOs) were examined for fumigant activity against immature and adult stages of Tephritidae flies. In addition to mortality, the percentage of main compounds of tested EOs is reported.

Species	Stage	Tested EO	Botanical Family	Main Constituents	Mortality Rates	References	Notes
***Anastrepha*** ***fraterculus***	Adult	*Baccharis* *dracunculifolia*	Asteraceae	β-pinene (22.69%); limonene (19.07%); nerolidol (8.08%); γ-elemene (7.80%); β-caryophyllene (6.17%); α-pinene (5.36%)	♂ 6.30 ± 0.27 days ♀ 6.76 ± 0.28 days	[46]	Mortality is expressed as the longevity of males and females after exposure to the EO
***Anastrepha*** ***fraterculus***	Adult	*Pinus* *elliottii*	Pinaceae	α-pinene (39.25%); β-pinene (34.79%); β-phellandrene (11.93%); limonene (9.31%)	♂ 9.02 ± 0.23 days ♀ 8.88 ± 0.24 days	[46]	Mortality is expressed as the longevity of males and females after exposure to the EO
***Anastrepha*** ***fraterculus***	Adult	*Cymbopogon* *citratus*	Poaceae	Purchased EO	62.5% on peach	[111]	EO dose 1% (*w*/*v*)
***Anastrepha*** ***fraterculus***	Adult	*Cymbopogon* *winterianus*	Poaceae	Purchased EO	80% on apple 100% on peach	[111]	EO dose 10% (*w*/*v*)
***Anastrepha*** ***fraterculus***	Adult	*Ruta* *graveolens*	Rutaceae	Purchased EO	Low mortality	[111]	EO dose 0.05% (*w*/*v*)
***Bactrocera*** ***dorsalis***	Adult	*Ocimum* *basilicum*	Lamiaceae	Purchased EO	LC_50_: 0.1–1%	[45]	
***Bactrocera*** ***dorsalis***	Adult	*Cymbopogon* *nardus*	Poaceae	Purchased EO	Low mortality	[130]	
***Bactrocera*** ***dorsalis***	Adult	*Eucalyptus* *camaldulensis*	Myrtaceae	Purchased EO	100% (after 12 h)	[130]	
***Bactrocera*** ***dorsalis***	Adult	*Eugenia* *caryophyllata*	Myrtaceae	Purchased EO	87.5% (after 72 h)	[130]	On day 15
***Bactrocera*** ***dorsalis***	Adult	*Ocimum* *basilicum*	Lamiaceae	Purchased EO	95% (after 72 h)	[130]	On day 15
***Bactrocera*** ***oleae***	Adult	*Mentha* × *piperita*	Lamiaceae	Linalool (40.4%); linalyl acetate (32.6%); α-terpineol (6.4%)	LC_50_: 0.27 μL/L air LC_90_: 0.45 μL/L air	[132]	
***Bactrocera*** ***oleae***	Adult	*Mentha* *pulegium*	Lamiaceae	Pulegone (77.3%); menthone (10.8%)		[132]	
***Bactrocera*** ***oleae***	Adult	*Mentha* *rotundifolia*	Lamiaceae	Menthone (28.5%); *iso*-menthone (19%); *neo*-menthol (10.4%)		[132]	
***Bactrocera*** ***oleae***	Adult	*Mentha* *spicata*	Lamiaceae	Carvone (54.1%); limonene (21.9%)	LC_50_: 0.22 μL/L air LC_90_: 0.33 μL/L air	[132]	
***Bactrocera*** ***oleae***	Adult	*Ammoides* *verticillata*	Apiaceae	Carvacrol (44.3%); limonene (19.3%); *p*-cymene (19.2%); γ-terpinene (11.1%)	LC_50_: <2 μL/L air	[142]	
***Bactrocera*** ***oleae***	Pupae	*Ammoides* *verticillata*	Apiaceae	Carvacrol (44.3%); limonene (19.3%); *p*-cymene (19.2%); γ-terpinene (11.1%)	LC_50_: 7.2 μL/L air air	[142]	
***Bactrocera*** ***oleae***	Larva	*Ammoides* *verticillata*	Apiaceae	Carvacrol (44.3%); limonene (19.3%); *p*-cymene (19.2%); γ-terpinene (11.1%)	LC_50_: 10.1 μL/L air air	[142]	
***Ceratitis*** ***capitata***	Adult	*Hyptis* *suaveolens*	Lamiaceae	Sabinene (34.0%); β-caryophyllene (11.2%); terpinolene (10.7%); β-pinene (8.2%)	LC_50_: 18.33 μL/L air air	[49]	
***Ceratitis*** ***capitata***	Adult	*Lavandula* *angustifolia*	Lamiaceae	Linalool (36.5%); linalyl acetate (14.4%); camphor (8.5%); 1,8-cineole (7.9%)	LC_50_: 9.08 μL/L air air	[49]	
***Ceratitis*** ***capitata***	Adult	*Rosmarinus* *officinalis*	Lamiaceae	1,8-cineole (34.3%); α-pinene (11.9%); borneol (10.0%)	LC_50_: 16.72 μL/L air	[49]	
***Ceratitis*** ***capitata***	Adult	*Thuja* *occidentalis*	Cupressaceae	δ-3-carene (33.2%); α-pinene (27.7%); cedrol (10.3%); terpinolene (5.7%)	LC_50_: 33.90 μL/L air air	[49]	
***Ceratitis*** ***capitata***	Adult	*Melaleuca* *alternifolia*	Myrtaceae	Terpinen-4-ol (35.1%); γ-terpinene (17.4%); α-terpinene (10.7%); 1,8-cineole (5.5%)	LC_50_: 2.24 μL/L air air	[48]	LC_50_ on *Psyttalia concolor*: 9.35 μL/L air
***Ceratitis*** ***capitata***	Adult	*Baccharis* *dracunculifolia*	Asteraceae	β-pinene (22.69%); limonene (19.07%); nerolidol (8.08%); γ-elemene (7.80%); β-caryophyllene (6.17%); α-pinene (5.36%)	♂ 7.23 ± 0.24 days ♀ 9.61 ± 0.22 days	[46]	Mortality is expressed as the longevity of males and females after the exposition to the EO
***Ceratitis*** ***capitata***	Adult	*Pinus elliottii*	Pinaceae	α-pinene (39.25%); β-pinene (34.79%); β-phellandrene (11.93%); limonene (9.31%)	♂ 4.92 ± 0.24 days ♀ 6.64 ± 0.29 days	[46]	Mortality is expressed as the longevity of males and females after the exposition to the EO
***Ceratitis*** ***capitata***	Adult	*Ocimum* *basilicum*	Lamiaceae	Purchased EO	LC_50_: 1–2.5%	[45]	
***Zeugodacus*** ***cucurbitae***	Adult	*Cymbopogon nardus*	Poaceae	Purchased EO	Low mortality	[130]	
***Zeugodacus*** ***cucurbitae***	Adult	*Ocimum* *basilicum*	Lamiaceae	Purchased EO	LC_50_: 1–2.5%	[45]	sub *Bactrocera*
***Zeugodacus*** ***cucurbitae***	Adult	*Eucalyptus* *camaldulensis*	Myrtaceae	Purchased EO	100% (after 12 h)	[130]	
***Zeugodacus*** ***cucurbitae***	Adult	*Eugenia* *caryophyllata*	Myrtaceae	Purchased EO	76.7% (after 72 h)	[130]	On day 15
***Zeugodacus*** ***cucurbitae***	Adult	*Ocimum* *basilicum*	Lamiaceae	Purchased EO	40.0% (after 72 h)	[130]	On day 15

♂ = males; ♀ = females; LC = lethal concentration.

**Table 3:** Terpenoids and phenylpropanoids were examined for fumigant action against immature and adult stages of Tephritidae flies.

Species	Stage	Tested Substance	Chemical Class	Mortality Rates	References	Notes
***Anastrepha*** ***fraterculus***	Egg	Citral	Monoterpenoid	LC_50_: 0.04 μL/cm^3^ air LC_90_: 0.16 μL/cm^3^ air	[141]	
***Anastrepha*** ***fraterculus***	Egg	Limonene	Monoterpene	LC_50_: 0.16 μL/cm^3^ air LC_90_: 0.27 μL/cm^3^ air	[141]	
***Bactrocera*** ***dorsalis***	Adult	Estragole	Phenylpropanoid	LC_50_: 1–2.5%	[45]	
***Bactrocera*** ***dorsalis***	Adult	Linalool	Monoterpenoid	LC_50_: 1–2.5%	[45]	
***Bactrocera*** ***dorsalis***	Adult	*trans*-Anethole	Phenylpropanoid	LC_50_: 0.1–1%	[45]	
***Ceratitis*** ***capitata***	Adult	Estragole	Phenylpropanoid	LC_50_: 0.75–1%	[45]	
***Ceratitis*** ***capitata***	Adult	Linalool	Monoterpenoid	LC_50_: 1–2.5%	[45]	
***Ceratitis*** ***capitata***	Adult	Methyl eugenol	Phenylpropanoid	LC_50_: 0.25–0.5%	[45]	
***Ceratitis*** ***capitata***	Adult	*trans*-Anethole	Phenylpropanoid	LC_50_: 0.75–1%	[45]	
***Ceratitis*** ***capitata***	Adult	*trans*-Anethole	Phenylpropanoid	logLC_50_: 0.2–0.3	[134]	Compound tested in M·cm^−3^, LC_50_ unit not provided in Figure 1
***Ceratitis*** ***capitata***	Adult	α-Pinene	Monoterpene	logLC_50_: 1.2–1.5	[134]	Compound tested in M·cm^−3^, LC_50_ unit not provided in Figure 1
***Ceratitis*** ***capitata***	Adult	Carvacrol	Monoterpenoid	logLC_50_: ~0.5	[134]	Compound tested in M·cm^−3^, LC_50_ unit not provided in Figure 1
***Ceratitis*** ***capitata***	Adult	Cinnamaldehyde	Phenylpropanoid	logLC_50_: ~0.4	[134]	Compound tested in M·cm^−3^, LC_50_ unit not provided in Figure 1
***Ceratitis*** ***capitata***	Adult	Cuminaldehyde	Phenylpropanoid	logLC_50_: 0.2–0.3	[134]	Compound tested in M·cm^−3^, LC_50_ unit not provided in Figure 1
***Ceratitis*** ***capitata***	Adult	Eugenol	Phenylpropanoid	logLC_50_: ~1	[134]	Compound tested in M·cm^−3^, LC_50_ unit not provided in Figure 1
***Ceratitis*** ***capitata***	Adult	Linalool	Monoterpenoid	logLC_50_: 0.5–0.7	[134]	Compound tested in M·cm^−3^, LC_50_ unit not provided in Figure 1
***Ceratitis*** ***capitata***	Adult	*p*-Cymene	Phenylpropanoid	logLC_50_: 1.5–1.8	[134]	Compound tested in M·cm^−3^, LC_50_ unit not provided in Figure 1
***Ceratitis*** ***capitata***	Adult	Terpineol	Monoterpenoid	logLC_50_: ~0.5	[134]	Compound tested in M·cm^−3^, LC_50_ unit not provided in Figure 1
***Zeugodacus*** ***cucurbitae***	Adult	Estragole	Phenylpropanoid	LC_50_: 1%	[45]	sub *Bactrocera*
***Zeugodacus*** ***cucurbitae***	Adult	Linalool	Monoterpenoid	LC_50_: 2.5–5%	[45]	sub *Bactrocera*
***Zeugodacus*** ***cucurbitae***	Adult	*trans*-Anethole	Phenylpropanoid	LC_50_: 0.75–1%	[45]	sub *Bactrocera*
***Zeugodacus*** ***cucurbitae***	Adult	Methyl eugenol	Phenylpropanoid	LC_50_: 0.25–0.5%	[45]	sub *Bactrocera*

LC = lethal concentration.

In conclusion, the susceptibility of true fruit flies to EOs and their main compounds varies according to life stage and delivery mode, similarly to what has been found for several species of stored-product pests [155]. From a practical point of view, fumigation toxicity may be considered of limited relevance for field application against tephritids, in contrast to ingestion toxicity, which is of practical importance for many “attract and kill” approaches.

#### 2.3.2. Topical/Contact Toxicity of Essential Oils and Their Main Compounds

In this paragraph, we reported the EOs toxicity towards tephritid flies when applied topically or in contact bioassays. Topical bioassays consist of the application of toxics on the insect body surface using a micro-applicator. For tephritids, a drop of the candidate insecticide is typically distributed on the thorax of adults [48,49]. On the other hand, contact bioassays generally consist of residual contact toxicity trials, in which a surface was treated with the putative insecticide [141]. The most studied plant families were Lamiaceae, Asteraceae, Myrtaceae, Apiaceae, and Rutaceae. According to the reviewed literature, the most studied species is *C. capitata* with 19 EOs, 4 monoterpenoids, and 3 mixtures tested, followed by *Bactrocera* (2 EOs and 7 compounds), *Z. cucurbitae* (1 EO and 2 compounds), and *Anastrepha* (2 EOs 2 compounds) [45,46,48,49] (Table 4 and Table 5).

Few studies evaluated the toxicity of some EOs either in fumigant, contact, and ingestion bioassays [48,49]. Unlike the results obtained through fumigation, all the EOs tested for topical toxicity, i.e., *Hyptis suaveolens* (L.) Kuntze (Lamiaceae), *L. angustifolia*, *M. alternifolia*, *R. officinalis*, and *Thuja occidentalis* L. (Cupressaceae), have a good insecticidal activity towards adults of *C. capitata* at 24 h [48,49]. *T. occidentalis* showed the lowest LD_50_ value (0.024 mL/fly), followed by *R. officinalis* (0.047 mL/fly), and *H. suaveolens* (0.066 mL/fly). The main constituents of *T. occidentalis* EO are δ-3-carene and α-pinene, widely known to be toxic to other arthropods [156]. For *M. alternifolia* EO, the toxicity was tested also on the parasitoid of *C. capitata*, the braconid wasp *P. concolor*, showing that this EO, when administrated topically, may cause higher mortality compared to fumigant bioassays [48]. These discrepancies may indicate the highest selectivity of the EO when administered by the fumigation technique.

Interestingly, citrus peel EOs are considered one of the plant mechanisms of protection against true fruit flies [124,157]. The toxicity of the citrus peel EOs is commonly associated with the presence of oxygenated and non-oxygenated terpenes such as limonene, α-pinene, β-pinene, linalool, and citral [124,141]. Directly exploring the role of citrus EOs main compounds, Papanastasiou et al. [124] highlighted that the toxicity of limonene, linalool, and α-pinene against both sexes of *C. capitata* is similar, though males seem more susceptible than females to these compounds. EOs containing α-pinene have been already proved to be toxic to *C. capitata* adults [49]. However, these results contrast with those reported by Papachristos et al. [126], who suggested that linalool and limonene are more toxic than α-pinene towards *C. capitata* larvae. The discrepancy may be attributed to the different delivery modes of the compounds, as well as to the developmental stages of the insect. Papanastasiou et al. [124] also investigated the impact of sub-lethal doses of limonene, suggesting that this compound may have a hormetic-like effect or an insecticidal one depending on the dosage. Limonene, together with α-pinene and β-pinene may be responsible for the highly toxic activity of *Baccharis dracunculifolia* DC. (Asteraceae) and *Pinus elliottii* Engelm. (Pinaceae) EOs on pupal mortality. In a study by Oviedo et al. [33], these EOs completely suppress the adult eclosion of *C. capitata* and less than 20% of *A. fraterculus* emerge. The efficacy of limonene as a toxin has also been reported by Ruiz et al. [141] in topical bioassays towards eggs and larvae of *A. fraterculus* and *C. capitata*. Interestingly, the eggs of *A. fraterculus* are more sensitive (citral LD_50_: 12.82 μL/mL; limonene LD_50_: 34.04 μL/mL) than those of *C. capitata* (citral LD_50_: 22.44 μL/mL; limonene LD_50_: 77.06 μL/mL). The higher sensitivity may be related to a difference of egg permeability. On the other hand, LD_50_ values resulted similar in larvicidal toxicity assays for all tested compounds [141]. Noteworthy, limonene is also a component of the male sex pheromone of both fruit flies [158,159] and acts as an attractant towards males and females of *C. capitata* [32].

Comparing the toxicity results with attractive/repellent ones, we can state that the biological activity of EOs is dose- and composition-dependent since changes in relative proportions of the same components substantially alter the insecticidal and olfactory activities of the EO itself. In this scenario, a study has reported interesting results about the exposition of males and females of *C. capitata* to EOs belonging to the plant genus *Tagetes* L. (Asteraceae) [113]. Among the tested EOs, *T. rupestris* Cabrera shows the best topical insecticidal activity towards both sexes but does not exert any attractive action for either males or females. Two other EOs, (i.e., *T. minuta* L. and *T. ternifolia* Kunth), though less toxic, can attract males and both sexes respectively [113]. The attractive nature of *T. minuta* and *T. ternifolia* might be explained by the presence of limonene and *p*-cymene in their formulations, both known for their attractiveness toward *C. capitata* adults [32,33]. The EOs of *Schinus polygama* (Cav.) Cabrera (Anacardiaceae), *Baccharis spartioides* (Hook. & Arn.) Gay (Asteraceae), *B. darwinii* Hook. & Arn. (Asteraceae), and *Azorella cryptantha* (Clos) Reiche (Apiaceae) have similar toxic properties to those extracted from *Tagetes* species [113,160,161,162]. As for *Tagetes* species, females of *C. capitata* are more sensitive towards *B. spartioides* EO than males (LD_50_♂__: 14.6 μg/fly; LD_50_♀__: 10.7μg/fly), whereas females exposed to *S. polygama* and *B. darwinii* EOs result more tolerant (*S. polygama* EO LD_50_♂__: 10.3 μg/fly; LD_50_♀__: 22.0 μg/fly; *B. darwinii* EO LD_50_♂__: 19.9 μg/fly LD_50_♀__: 31.0 μg/fly) [160,161]. Similarly to *B. dracunculifolia*, the toxicity of *B. darwinii* EO might be related to the presence of limonene, but minor components, such as thymol and terpinene-4-ol, might modulate the whole efficacy of the EO which varies also according to the pest species, life cycle stage, and sex. Interestingly, investigating the toxicity of *A. cryptantha* EO toward both sexes of *C. capitata*, López et al. [162] noticed how differences in altitude, climatic and edaphic conditions influence the chemical components and subsequently the toxic activity of EOs.

Mixing EOs can promote synergism between molecules, increasing the toxic effect [163,164,165,166], and helping to prevent or delay the development of resistance by pests [167]. Among the tested mixtures, topical applications of CEL (a mixture of EOs of *Cymbopogon citratus* (DC.) Stapf (Poaceae), *Cedrus atlantica* (Endl.) Manetti ex Carriere (Pinaceae), and *Corymbia citriodora* (Hook.) K. D. Hill & L. A. S. Johnson (Myrtaceae)) cause high mortality on adults of *C. capitata*, although resulting harmless to the parasitoid *P. concolor* [131,168]. CEL toxicity may be due to the presence of monoterpenoids (i.e., *E*-citral, *Z*-citral, and citronellal), which are known to interfere with different sites of action and to affect pupation, adult emergence, deformation, oviposition, adult longevity, and ovarian development [131,148].

**Table 4:** Essential oils (EOs) were examined for topical/contact action against immature and adult stages of Tephritidae flies. In addition to mortality, the percentage of main compounds of tested EOs is reported.

Species	Stage	Tested EO	Botanical Family	Main Constituents	Mortality Rates	References	Notes
***Anastrepha fraterculus***	Pupa	*Baccharis* *dracunculifolia*	Asteraceae	β-pinene (22.69%); limonene (19.07%); nerolidol (8.08%); γ-elemene (7.80%); β-caryophyllene (6.17%)	Adult emergence 21%	[33]	
***Anastrepha fraterculus***	Pupa	*Pinus* *elliottii*	Pinaceae	α-pinene (39.25%); β-pinene (34.79%); β-phellandrene (11.93%); limonene (9.31%)	Adult emergence 15%	[33]	
***Bactrocera oleae***	Larva	*Mentha* *pulegium*	Lamiaceae	Pulegone (75.7%); menthone (10.1%)	LD_50_: 1.79 μL/mL	[169]	
***Bactrocera oleae***	Larva	*Salvia* *fruticosa*	Lamiaceae	1,8-cineole (52.5%); α-thujone (8.3%); β-thujone (3.1%); camphor (0.9%)	LD_50_: 0.22 μL/mL	[169]	
***Ceratitis*** ***capitata***	Adult	*Baccharis* *spartoides*	Asteraceae	α-phellandrene (44.5%); sabinene (20.7%); β-pinene (15.9%)	LD_50_: 14.60 μg/fly ♂ LD_50_: 10.7 μg/fly ♀	[160]	
***Ceratitis*** ***capitata***	Adult	*Schynus* *polygama*	Anacardiaceae	δ-cadinene (7.8%); γ-cadinene (5.3%); β-caryophyllene (5.1%); *trans*-muurola-4(14),5-diene (4.7%); terpinene (4.6%); α-pinene (4.2%)	LD_50_: 10.3 μg/fly ♂ LD_50_: 22.0 μg/fly ♀	[160]	
***Ceratitis*** ***capitata***	Adult	*Hyptis* *suaveolens*	Lamiaceae	Sabinene (34.0%); β-caryophyllene (11.2%); terpinolene (10.7%); β-pinene (8.2%)	LD_50_: 0.066 μL/fly	[49]	
***Ceratitis*** ***capitata***	Adult	*Lavandula* *angustifolia*	Lamiaceae	Linalool (36.5%); linalyl acetate (14.4%); camphor (8.5%); 1,8-cineole (7.9%)	LD_50_: 0.017 μL/fly	[49]	
***Ceratitis*** ***capitata***	Adult	*Rosmarinus* *officinalis*	Lamiaceae	1,8-cineole (34.3%); α-pinene (11.9%); borneol (10.0%)	LD_50_: 0.047 μL/fly	[49]	
***Ceratitis*** ***capitata***	Adult	*Thuja* *occidentalis*	Cupressaceae	δ-3-carene (33.2%); α-pinene (27.7%); cedrol (10.3%); terpinolene (5.7%)	LD_50_: 0.024 μL/fly	[49]	
***Ceratitis*** ***capitata***	Adult	*Melaleuca* *alternifolia*	Myrtaceae	Terpinen-4-ol (35.1%); γ-terpinene (17.4%); α-terpinene (10.7%); 1,8-cineole (5.5%)	LD_50_: 0.117 μL/cm^2^	[48]	LD_50_ on *Psyttalia* *concolor*: 0.147 μL/cm^2^
***Ceratitis*** ***capitata***	Adult	*Baccharis* *darwinii*	Asteraceae	Limonene (47.1%); thymol (8.1%); sabinene (5.7%); myrcene (3.6%); α-pinene (4.6%); α-terpineol (3.7%)	LD_50_: 19.9 μg/fly ♂ LD_50_: 30 μg/fly ♀	[161]	
***Ceratitis*** ***capitata***	Adult	*Tagetes* *minuta*	Asteraceae	*cis*-tagetone (62.4%); *trans*-β-ocimene (16.2%); dihydrotagetone (10.3%)	LD_50_: 18.32 μg/fly ♂ LD_50_: 14.74 μg/fly ♀	[113]	
***Ceratitis*** ***capitata***	Adult	*Tagetes* *rupestris*	Asteraceae	*trans*-ocimenone (39.3%); *trans*-tagetone (24.4%); *cis*-β-ocimene (6.1%); *cis*-ocimenone (5.9%)	LD_50_: 14.50 μg/fly ♂ LD_50_: 5.69 μg/fly ♀	[113]	
***Ceratitis*** ***capitata***	Adult	*Tagetes* *ternifolia*	Asteraceae	*cis*-tagetone (31.0%); *cis*-β-ocimene (15.4%); *trans*-ocimenone (15.4%); *cis*-ocimenone (14.5%); *trans*-tagetone (10.3%); dihydrotagetone (6.5%)	LD_50_: 19.97 μg/fly ♂ LD_50_: 16.17 μg fly ♀	[113]	
***Ceratitis*** ***capitata***	Adult	*Azorella* *cryptantha*	Apiaceae	α-pinene (21.9%); α-thujene (12.5%); cadinene (8.6%); sabinene (6.4%); δ-*trans*-β-guaiene (6.2%)	LD_50_: 2.60 μg/fly ♂ LD_50_: 9.54 μg/fly ♀	[162]	The plant species has been collected in Bauchaceta (Argentina)
***Ceratitis*** ***capitata ***	Adult	*Azorella* *cryptantha*	Apiaceae	α-thujene (5.7%); α-pinene (9.6%); β-pinene (5.9%); γ-cadinene (4.0%); δ-cadinene (6.3%)	LD_50_: 10.78 μg/fly ♂ LD_50_: 8.39 μg/fly ♀	[162]	The plant species has been collected in Aqua Negra (Argentina)
***Ceratitis*** ***capitata***	Pupa	*Baccharis* *dracunculifolia*	Asteraceae	β-pinene (22.69%); limonene (19.07%); nerolidol (8.08%); γ-elemene (7.80%); β-caryophyllene (6.17%); α-pinene (5.36%)	Adult emergence 0%	[33]	
***Ceratitis*** ***capitata***	Pupa	*Pinus* *elliottii*	Pinaceae	α-pinene (39.25%); β-pinene (34.79%); β-phellandrene (11.93%); limonene (9.31%)	Adult emergence 0%	[33]	
***Ceratitis*** ***capitata***	Adult	*Amyris* *balsamifera*	Rutaceae	Purchased EO	LD_50_: 0.014 μL/fly ♂ LD_50_: 0.026 μL/fly ♀	[131]	
***Ceratitis*** ***capitata***	Adult	*Cedrus* *atlantica*	Pinaceae	Purchased EO	LD_50_: 0.012 μL/fly ♂ LD_50_: 0.015 μL/fly ♀	[131]	
***Ceratitis*** ***capitata ***	Adult	*Corymbia* *citriodora*	Myrtaceae	Purchased EO	LD_50_: 0.032 μL/fly ♂ LD_50_: 0.033 μL/fly ♀	[131]	
***Ceratitis*** ***capitata***	Adult	*Cymbopogon* *citratus*	Poaceae	Purchased EO	LD_50_: 0.014 μL/fly ♂ LD_50_: 0.022 μL/fly ♀	[131]	
***Ceratitis*** ***capitata***	Adult	*Pelargonium* *Graveolens*	Geraniaceae	Purchased EO	LD_50_: 0.029 μL/fly ♂ LD_50_: 0.029 μL/fly ♀	[131]	
***Ceratitis*** ***capitata***	Adult	CEL (*C. atlantica +* *C. citriodora +* *C. citratus)*	Pinaceae + Myrtaceae + Poaceae	Purchased EO	LD_50_: 0.018 μL/fly ♂ LD_50_: 0.018 μL/fly ♀	[131]	Additive effect
***Ceratitis*** ***capitata***	Adult	SLD (*A. balsamifera +* *C. citratus +* *C. atlantica)*	Rutaceae + Poaceae + Pinaceae	Purchased EO	LD_50_: 0.016 μL/fly ♂ LD_50_: 0.018 μL/fly ♀	[131]	Additive effect
***Ceratitis*** ***capitata***	Adult	GES (*P. graveolens +* *C. citriodora +* *A. balsamifera)*	Geraniaceae + Myrtaceae + Rutaceae	Purchased EO	LD_50_: 0.015 μL/fly ♂ LD_50_: 0.029 μL/fly ♀	[131]	Additive effect
***Zeugodacus cucurbitae***	Adult	*Peperomia* *borbonensis*	Piperaceae	Decanal (7.3%); δ-elemene (4.9%); myristicin (39.5%); elemicin (26.6%)	LD_50_: 0.23 μg/cm^2^ LD_90_: 0.34 μg/cm^2^	[170]	sub *Bactrocera*

♂ = males, ♀ = females, LD = lethal dose.

**Table 5:** Terpenoids and phenylpropanoids were examined for contact action against immature and adult stages of Tephritidae flies.

Species	Stage	Tested Substance	Chemical Class	Mortality Rates	References	Notes
***Anastrepha*** ***fraterculus***	Egg	Citral	Monoterpenoid	LD_50_: 12.82 μL/mL LD_90_: 16.79 μL/mL	[141]	
***Anastrepha*** ***fraterculus***	Larva	Citral	Monoterpenoid	LD_50_: 1.62 μL/mL LD_90_: 4.98 μL/mL	[141]	
***Anastrepha*** ***fraterculus***	Egg	Limonene	Monoterpene	LD_50_: 34.04 μL/mL LD_90_: 80.37 μL/mL	[141]	
***Anastrepha*** ***fraterculus***	Larva	Limonene	Monoterpene	LD_50_: 0.84 μL/mL LD_90_: 23.93 μL/mL	[141]	
***Bactrocera*** ***oleae***	Larva	1,8-Cineole	Monoterpenoid	LD_50_: 0.50 μL/mL	[169]	
***Bactrocera*** ***oleae***	Larva	Camphor	Monoterpenoid	LD_50_: 1.45 μL/mL	[169]	
***Bactrocera*** ***oleae***	Larva	Menthone	Monoterpenoid	LD_50_: 0.13 μL/mL	[169]	
***Bactrocera*** ***oleae***	Larva	Pulegone	Monoterpenoid	LD_50_: 0.09 μL/mL	[169]	
***Bactrocera*** ***oleae***	Larva	Thujone	Monoterpenoid	LD_50_: 0.82 μL/mL	[169]	
***Bactrocera*** ***zonata***	Larva	(1*R*, 2*S*, 5*R*)- Menthol	Monoterpenoid	LD_50_: <20 mg/kg	[148]	After 72 h
***Bactrocera*** ***zonata***	Larva	(*R*)-Camphor	Monoterpenoid	LD_50_: 23.68 mg/kg	[148]	After 72 h
***Bactrocera*** ***zonata***	Larva	(*R*)-carvone	Monoterpenoid	LD_50_: <20 mg/kg	[148]	After 72 h
***Ceratitis*** ***capitata***	Egg	Citral	Monoterpenoid	LD_50_: 22.44 μL/mL LD_90_: 41.76 μL/mL	[141]	
***Ceratitis*** ***capitata***	Larva	Citral	Monoterpenoid	LD_50_: 3.18 μL/mL LD_90_: 7.69 μL/mL	[141]	
***Ceratitis*** ***capitata***	Egg	Limonene	Monoterpene	LD_50_: 77.06 μL/mL LD_90_: 119.64 μL/mL	[141]	
***Ceratitis*** ***capitata***	Larva	Limonene	Monoterpene	LD_50_: 2.30 μL/mL LD_90_: 2.28 μL/mL	[141]	
***Ceratitis*** ***capitata***	Adult	Limonene	Monoterpene	LD_50_: 8.34 nL/fly ♂ LD_90_: 44.01 nL/fly ♂ LD_50_: 31.72 nL/fly ♀ LD_90_: 155.77 nL/fly ♀	[124]	Diet yeast + sugar
***Ceratitis*** ***capitata***	Adult	Linalool	Monoterpenoid	LD_50_: 10.37 nL/fly ♂ LD_90_: 57.05 nL/fly ♂ LD_50_: 49.39 nL/fly ♀ LD_90_: 210.42 nL/fly ♀	[124]	Diet yeast + sugar
***Ceratitis*** ***capitata***	Adult	α-Pinene	Monoterpene	LD_50_: 7.71 nL/fly ♂ LD_90_: 30.34 nL/fly ♂ LD_50_: 17.20 nL/fly ♀ LD_90_: 71.32 nL/fly ♀	[124]	Diet yeast + sugar
***Zeugodacus*** ***cucurbitae***	Adult	Elemicin	Phenylpropanoid	<40%	[170]	Tested separately according to the ratio found in the EO. Reported sub *Bactrocera*
***Zeugodacus*** ***cucurbitae***	Adult	Myristicin	Phenylpropanoid	<40%	[170]	Tested separately according to the ratio found in the EO. Reported sub *Bactrocera*

♂ = males, ♀ = females, LD = lethal dose.

#### 2.3.3. Ingestion Toxicity of Essential Oils and Their Main Compounds

In ingestion bioassays, the toxics are given to insects incorporated into sugar or a protein/food bait to encourage feeding. The insecticidal activity of EOs by ingestion has been evaluated for 25 EOs, 16 compounds, and 1 mixture of EOs. The most studied plant families were Lamiaceae, Asteraceae, Myrtaceae, Apiaceae, and Rutaceae. According to the reviewed literature, the most studied plant is *C. capitata* with 19 EOs, 16 terpenes, and phenylpropanoids investigated, followed by *Bactrocera* (7 EOs), and *Anastrepha* (5 EOs) [5,33,47,126,171] species (Table 6 and Table 7).

*Thymus* (Lamiaceae) EOs have been tested by ingestion on *C. capitata* adults. Amongst *Thymus* spp., the most toxic EO is *T. capitatus* (L.) Hoffmanns. & Link one (64.2%), followed by *T. albicans* Hoffmanns. & Link (15–20 %), and *T. herba-barona* Loisel. (14.2%) [172,173]. However, at a higher dose, the EO of *T. herba-barona* causes the death of 91% of *C. capitata* adults [173]. The higher toxicity of *T. capitatus* compared to *T. herba-barona* is due to the greater presence of carvacrol, a compound completely absent from *T. albicans* EO, although this EO is rich in 1,8-cineole [172,173]. Carvacrol has a cytotoxic effect when absorbed and stored in the tissues [173]. Many EOs of the genus *Thymus* have thymol as one of their main components, which is absent or just present in traces in the above-mentioned EOs. Thymol is responsible for the toxic activity of the EO extracted by *T. vulgaris* L. towards *Anastrepha ludens* adults [174]. In this plant, thymol may interact synergistically with other compounds such as α-terpineol and linalool. Linalool is also a component of basil EO, whose insecticidal activity against *A. ludens* adults is however lower compared with *T. vulgaris* (LD_50_: 8050 ppm vs. 5347 ppm, respectively) [174]. The toxicity of other constituents, such as linalool, α-terpineol, terpinene-4-ol, and neral, has also been noted on *C. capitata* larvae, with an LD_50_ value lower than 5 μL/g of food [126].

The biological activity of EOs is closely dependent on the tested insect species. Experiments conducted using the same EO may show variable results when different fly species are used. For instance, the EOs of *H. suaveolens* and *Trachyspermum ammi* (L.) Sprague (Apiaceae) are more toxic to *B. oleae* than *C. capitata* [5,47,49,171], while *R. officinalis* EO displays higher toxicity towards *C. capitata* than *B. oleae* [5,49]. However, the latter results contrast with those obtained by Sanna-Passino et al. [173], who observed a low efficacy of both *R. officinalis* and *Salvia officinalis* L. (Lamiaceae) EOs against *C. capitata* adults. These discrepancies in biological activity may be related to differences in insect strains, as well as to the chemical composition of the tested EOs [172].

Among the EOs tested by ingestion on *C. capitata* adults, the EO of *Carlina acaulis* L. (Asteraceae) appears to be one of the most toxic [171]. The major compound (>90%) of this EO is carlina oxide, which is already known as an effective insecticide, and whose mode of action is partially linked to AChE inhibition [175]. Furthermore, aromatic polyacetylenes, as carlina oxide, can cause phototoxicity in insects [176] and modulate the GABA-A receptors [177].

Relatively few citrus EOs have been tested in ingestion bioassays. Interestingly, citrus EOs seem to be involved in various aspects of the life of true fruit flies; they can reduce oviposition [126,178], attract sexually mature males [34,179], and tephritid males exposed to these EOs can acquire a significant mating advantage over unexposed males [56,180]. Among the citrus EOs, *C. limon* EO resulted less toxic by ingestion to *C. capitata* larvae than *C. sinensis* and *C. aurantium* L. EOs [126]. Further studies are needed to shed light on the role of citrus EOs on the biology of *C. capitata* and other tephritids.

**Table 6:** Essential oils (EOs) were examined for ingestion toxicity against immature and adult stages of Tephritidae flies. In addition to mortality, the percentage of main compounds of tested EOs is reported.

Species	Stage	Tested EO	Botanical Family	Main Constituents	Mortality Rates	References	Notes
***Anastrepha*** ***fraterculus***	Adult	*Baccharis* *dracunculifolia*	Asteraceae	β-pinene (22.69%); limonene (19.07%); nerolidol (8.08%); γ-elemene (7.80%); β-caryophyllene (6.17%)	Living adults 58.67%	[33]	Results about *A. fraterculus* were combined with the *C. capitata* ones
***Anastrepha*** ***fraterculus***	Adult	*Pinus* *elliottii*	Pinaceae	α-pinene (39.25%); β-pinene (34.79%); β-phellandrene (11.93%); limonene (9.31%)	Living adults 70.33%	[33]	Results about *A. fraterculus* were combined with the *C. capitata* ones
***Anastrepha*** ***ludens***	Adult	*Eugenia* *caryophyllata*	Myrtaceae	Eugenol (77.58%); acetyl eugenol (10.99%); β-caryophyllene (6.22)	LD_50_: 3529 ppm LD_90_: 7763 ppm	[174]	
***Anastrepha*** ***ludens***	Adult	*Ocimum* *basilicum*	Lamiaceae	Estragole (72.64%); linalool (16.65%)	LD_50_: 8050 ppm LD_90_: 25,846 ppm	[174]	
***Anastrepha*** ***ludens***	Adult	*Thymus* *vulgaris*	Lamiaceae	*p*-cymene (32.49%); α-terpineol (12.58%); linalool (5.29%)	LD_50_: 5347 ppm LD_90_: 18,113 ppm	[174]	
***Bactrocera*** ***oleae***	Adult	*Hyptis* *suaveolens*	Lamiaceae	Sabinene (19.5%); β-caryophyllene (16.2%); terpinen-4-ol (7.7%); terpinolene (7.4%); β-pinene (6.7%)	LD_50_: 4922 ppm	[5]	
***Bactrocera*** ***oleae***	Adult	*Lavandula* *angustifolia*	Lamiaceae	Linalool (39.5%); linalyl acetate (18.2%); camphor (9.7%); 1,8-cineole (6.5%); borneol (6.6%)	LD_50_: 6271 ppm	[5]	
***Bactrocera*** ***oleae***	Adult	*Rosmarinus* *officinalis*	Lamiaceae	1,8-cineole (18.6%); α-pinene (15.6%); camphor (15.3%); borneol (9.2%); verbenone (8.2%)	LD_50_: 5107 ppm	[5]	
***Bactrocera oleae***	Adult	*Ocimum* *gratissimum*	Lamiaceae	Thymol (57.0%); *p*-cymene (12.4%); γ-terpinene (6.9%)	LD_50_: 925 ppm LD_90_: 6365 ppm	[47]	
***Bactrocera*** ***oleae***	Adult	*Pimpinella* *anisum*	Apiaceae	*trans*-anethole (98.3%)	LD_50_: 771 ppm LD_90_: 1981 ppm	[47]	
***Bactrocera*** ***oleae***	Adult	*Thymbra* *spicata*	Lamiaceae	Carvacrol (41.4%); *p*-cymene (41.2%); γ-terpinene (5.5%); thymol (5.2%)	LD_50_: 2509 ppm LD_90_: 12,519 ppm	[47]	
***Bactrocera*** ***oleae***	Adult	*Trachyspermum* *ammi*	Apiaceae	Thymol (58.3%); *p*-cymene (24.7%); γ-terpinene (14.2%)	LD_50_: 633 ppm LD_90_: 2131 ppm	[47]	
***Ceratitis*** ***capitata***	Adult	*Hyptis* *suaveolens*	Lamiaceae	Sabinene (34.0%); β-caryophyllene (11.2%); terpinolene (10.7%); β-pinene (8.2%)	LD_50_: 13,041 ppm	[49]	
***Ceratitis*** ***capitata***	Adult	*Lavandula* *angustifolia*	Lamiaceae	Linalool (36.5%); linalyl acetate (14.4%); camphor (8.5%); 1,8-cineole (7.9%)	LD_50_: 6860 ppm	[49]	
***Ceratitis*** ***capitata***	Adult	*Rosmarinus* *officinalis*	Lamiaceae	1,8-cineole (34.3%); α-pinene (11.9%); borneol (10.0%)	LD_50_: 3664 ppm	[49]	
***Ceratitis*** ***capitata***	Adult	*Thuja* *occidentalis*	Cupressaceae	δ-3-carene (33.2%); α-pinene (27.7%); cedrol (10.3%); terpinolene (5.7%)	LD_50_: 5371 ppm	[49]	
***Ceratitis*** ***capitata***	Adult	*Melaleuca* *alternifolia*	Myrtaceae	Terpinen-4-ol (35.1%); γ-terpinene (17.4%); α-terpinene (10.7%); 1,8-cineole (5.5%)	LD_50_: 0.269% (*w*/*v*)	[48]	LD_50_ on *Psyttalia concolor*: 0.639% *w*/*w*
***Ceratitis*** ***capitata***	Adult	*Carlina* *acaulis*	Asteraceae	Carlina oxide (97.7%)	LD_50_: 1094 ppm LD_90_: 3082 ppm	[171]	Sublethal effect on aggressive behavior
***Ceratitis*** ***capitata***	Adult	*Trachyspermum* *ammi*	Apiaceae	Thymol (64.7%); *p*-cymene (17.0%); γ-terpinene (14.8%)	LD_50_: 3969 ppm LD_90_: 8200 ppm	[171]	Sublethal effect on aggressive behavior
***Ceratitis*** ***capitata***	Larva	*Teucrium* *leucocladum*	Lamiaceae	Patchouli alcohol (31.24%); β-pinene (12.66%); α-pinene (10.99%); cedrol (10.3%)	LD_50_: 24 ppm	[181]	
***Ceratitis*** ***capitata***	Adult	*Mentha* *pulegium*	Lamiaceae	Pulegone (27.3%); menthol (22.0%); menthone (14.0%); *iso*-menthone (14.0%)	>95% of adults	[172]	After 48 h, the emulsion contained 0.25% (*w*/*v*) of EO
***Ceratitis*** ***capitata***	Adult	*Thymbra* *capitata*	Lamiaceae	1,8-cineole (68.0%)	<35% of adults	[172]	After 48 h, the emulsion contained 0.25% (*w*/*v*) of EO
***Ceratitis*** ***capitata***	Adult	*Thymus* *albicans*	Lamiaceae	Carvacrol 82%	15–20% of adults	[172]	After 48 h, the emulsion contained 0.25% (*w*/*v*) of EO
***Ceratitis*** ***capitata***	Adult	*Baccharis* *dracunculifolia*	Asteraceae	β-pinene (22.69%); limonene (19.07%); nerolidol (8.08%); γ-elemene (7.80%); β-caryophyllene (6.17%)	Living adults 58.67%	[33]	Results about *C. capitata* were combined with the *A. fraterculus* ones
***Ceratitis*** ***capitata***	Adult	*Pinus* *elliottii*	Pinaceae	α-pinene (39.25%); β-pinene (34.79%); β-phellandrene (11.93%); limonene (9.31%)	Living adults 70.33%	[33]	Results about *C. capitata* were combined with the *A. fraterculus* ones
***Ceratitis*** ***capitata***	Larva	*Citrus* *aurantium*	Rutaceae	Limonene (96.7%)	>99% of adults	[126]	Dose 13 mL/g
***Ceratitis*** ***capitata***	Larva	*Citrus* *limon*	Rutaceae	Limonene (74.3%); γ-terpinene (6.4%); β-pinene (7.0%)	>99% of adults	[126]	Dose 16.5 mL/g
***Ceratitis*** ***capitata***	Larva	*Citrus* *sinensis*	Rutaceae	Limonene (97.4%)	>99% of adults	[126]	Dose 13 mL/g
***Ceratitis*** ***capitata***	Adult	*Rosmarinus* *officinalis*	Lamiaceae	α-pinene (33.95); 1,8-cineole (11.24%); bornyl acetate (7.80%); camphene (7.51%); farnesol (6.02%)	Low activity	[173]	After 72 h
***Ceratitis*** ***capitata***	Adult	*Salvia* *officinalis*	Lamiaceae	Camphor (26.85%); α-thujone (23.00%); 1,8-cineole (11.82%); camphene (5.80%)	Low activity	[173]	After 72 h
***Ceratitis*** ***capitata***	Adult	*Thymus* *capitatus*	Lamiaceae	Carvacrol (68.91%); γ-terpinene (6.33%); *p*-cymene (6.17%); β-caryophyllene (5.20%)	LD_50_: 93.0% (*w*/*w*)	[173]	After 72 h
***Ceratitis*** ***capitata***	Adult	*Thymus* *herba barona*	Lamiaceae	Carvacrol (44.59%); *p*-cymene (5.97%); γ-terpinene (5.56%); borneol (5.39%)	LD_50_: 91% (*w*/*w*)	[173]	After 72 h

LD = lethal dose.

**Table 7:** Terpenoids and phenylpropanoids were examined for ingestion action against immature and adult stages of Tephritidae flies.

Species	Stage	Tested Substance	Chemical Class	Mortality Rates	References
***Ceratitis capitata***	Larva	α-Terpineol	Monoterpenoid	The LD_50_ value is reported only graphically	[126]
***Ceratitis capitata***	Larva	(+)-β-Pinene	Monoterpene	The LD_50_ value is reported only graphically	[126]
***Ceratitis capitata***	Larva	Citral	Monoterpenoid	The LD_50_ value is reported only graphically	[126]
***Ceratitis capitata***	Larva	Geranyl acetate	Monoterpenoid	The LD_50_ value is reported only graphically	[126]
***Ceratitis capitata***	Larva	γ-Terpinene	Monoterpene	The LD_50_ value is reported only graphically	[126]
***Ceratitis capitata***	Larva	Linalool	Monoterpenoid	The LD_50_ value is reported only graphically	[126]
***Ceratitis capitata***	Larva	Linalyl acetate	Monoterpenoid	The LD_50_ value is reported only graphically	[126]
***Ceratitis capitata***	Larva	Myrcene	Monoterpene	9.6 μL/g food	[126]
***Ceratitis capitata***	Larva	Neryl acetate	Monoterpenoid	The LD_50_ value is reported only graphically	[126]
***Ceratitis capitata***	Larva	*R*-(+)-limonene	Monoterpene	6.2 μL/g food	[126]
***Ceratitis capitata***	Larva	*S*-(−)-limonene	Monoterpene	7 μL/g food	[126]
***Ceratitis capitata***	Larva	Terpinen-4-ol	Monoterpenoid	The LD_50_ value is reported only graphically	[126]
***Ceratitis capitata***	Larva	(−)-*trans*-Caryophyllene	Sesquiterpene	8.3 μL/g food	[126]
***Ceratitis capitata***	Larva	(+)-Valencene	Sesquiterpene	10.4 μL/g food	[126]
***Ceratitis capitata***	Larva	(−)-α-Pinene	Monoterpene	The LD_50_ value is reported only graphically	[126]
***Ceratitis capitata***	Larva	(+)-α-Pinene	Monoterpene	The LD_50_ value is reported only graphically	[126]

## 3. Mechanisms of Action of Essential Oils

Information about the mechanisms of action of EOs is still fragmentary. Since EOs are complex mixtures, in some cases of hundreds of constituents, it is difficult to outline a unique mode of action for all of them. Furthermore, the chemical composition of an EO may also vary depending on the plant genetic pool or abiotic factors influencing plant development, such as temperature, water availability, altitude, and soil fertility [182,183,184]. On the other hand, a deeper understanding of their spectrum of action on target insect species could be crucial for the development of new biopesticides based on EOs [185].

EOs can exert neurotoxicity by inactivating AChE, by modulating the octopaminergic system and GABA receptors [20,25]. Some EO constituents may operate as competitive inhibitors of AChE, an enzyme involved in neuro-neuronal and neuromuscular junctions in both insects and mammals [185]. In this case, EO compounds attach to the active sites of AChE, preventing the binding of the neurotransmitter acetylcholine and decreasing its availability while the maximal activity of the enzyme remains unchanged. Other molecules from EOs act as uncompetitive inhibitors [186,187,188,189,190,191]; in this case, they do not bind to the active site of AChE but they allosterically alter its action by attaching to a different site. Consequently, the maximum activity of the enzyme decreases [185]. Monoterpenoids, among the major compounds of the EOs, operate as AChE inhibitors [128,189], even if they act as competitive inhibitors only at relatively high concentrations [192,193] and their inhibitory action is quickly reversible [194]. Although major compounds of the EOs are considered AChE inhibitors, the neurotoxic action of EOs may involve other targets, such as GABA receptors [185]. Signal transmission at the synaptic level is determined by the opening of chlorine channels, allowing the chlorine ions to flow into the neuron, causing a change in its membrane potential and eventually hyperpolarization. The opening of these channels is in turn determined by the binding of GABA to specific transmembrane receptors [195]. The chlorine channels are the targets of some EO compounds, which stabilize the non-conductive conformations of these channels. EO constituents bind to the insect’s GABA receptors, either decreasing or increasing the Cl^-^ influx into the neurons, eventually killing the insect by causing an over-excitation or an inhibition of the nervous system [196]. According to a study conducted on American cockroach (*Periplaneta americana* (L.)), three monoterpenoids (i.e., carvacrol, pulegone, and thymol) enhanced the binding of [3H]-TBOB to membranes of the insect’s neuronal cells and increased the Cl^-^ uptake in insect membrane. The hypothesis that these monoterpenoids are allosteric modulators of GABA receptors is then supported [196]. In addition, thymol potentiates GABA receptors through an unidentified binding site [152], while the silphinenes (i.e., tricyclic sesquiterpenes) antagonize the action of GABA on insect neurons [197].

Lastly, another target of EOs is octopamine (OA), an invertebrate multifunctional molecule equivalent to noradrenaline of vertebrates [185]. Acting as a neurotransmitter, a neurohormone, and a neuromodulator [198,199,200], OA is present in the nervous system, neuroendocrine cells, and hemolymph [201]. Octopamine plays an essential role in the insect stress response, aggressive behavior, and social behavior [202,203], and is also known as the insect “fight or flight” hormone [204]. In addition, this molecule is a part of the arousal system which prepares an insect for vigorous activity [205]. OA binds to a specific G protein-coupled membrane receptor (GPCR), leading to the activation of the enzyme adenylyl cyclase. This enzyme transforms ATP in cAMP. An increase of cAMP leads to the activation of the protein kinase A (PKA), an enzyme that phosphorylates several different enzymes and receptors, modulating their activity; cAMP activates, also, the phospholipase C, leading to a rise in the intracellular level of Ca^2+^ and activation of calcium-dependent protein kinase C (PKC). This set of cascading changes inside cells results in a modification of insect behavior and response to external stimuli [185,206]. Mimicking the OA, EO components can interact with OA receptors, acting as OA agonists. Causing an increase in cAMP and intracellular Ca^2+^ levels, they can induce the activation of PKA and PKC and the phosphorylation of many proteins (including ion channels, enzymes, and receptors) [207]. EO constituents, such as eugenol and α-terpineol, induce an increase in cAMP levels, while others such as geraniol and citral decrease them. All of them reduce the binding of [3H]-OA to its receptors [150]. Since the presence of OA is negligible and no octopamine receptors are found in mammals, the agonist activity against OA receptors represents an interesting target for studies on neurotoxic effects of EOs to develop low-impact biopesticides for pest control in IPM programs.

## 4. Tephritid and Essential Oils: Real-World Applications and Challenges

Given their properties, EOs can be considered promising active principles for plant protection, as well as for food industry, human and animal health protection [20,30].

Concerning tephritids, to date, several studies have been carried out on the use of EOs to implement the Sterile Insect Technique (SIT), whose success relies on the ability of the sterile males to compete with the wild ones and induce sterility in wild females [208,209,210]. After sterilization, however, the competitiveness of the males is generally reduced, thus affecting the outcome of the SIT [211]. EOs and their main compounds boost the competitiveness of sterile males of *B. correcta*, *B. dorsalis*, and *Bactrocera philippinensis* Drew & Hancock, increasing their mating success and, consequently, the whole efficacy of SIT [208,209,210,212]. Albeit, possible undesirable and dose-dependent effects should be considered [213]; for instance, *B. dorsalis* males need more time to recover after being exposed to ME, which translates into a decrease in competitiveness [214].

Among EOs, ginger oil (GRO), rich in *α*-copaene, has been deeply investigated as a pre-release treatment for SIT programs with *C. capitata* males. As reported by Shelly and McInnis [215], GRO-treated sterile males of *C. capitata* obtained a higher mating percentage compared with untreated males. Taking it to a wider scale, Shelly et al. [216] reported that *Coffea arabica* L. (Rubiaceae) berries collected in plots with GRO-exposed males were less infested by *C. capitata* eggs. The use of GRO as pre-release treatment is then recommended in SIT programs [36]. Other EOs as *C. limon*, *S. polygama,* and the monoterpene limonene have also been suggested as a pre-release treatment for *A. fraterculus* sterile males since laboratory bioassays highlighted that the mating success of males of *A. fraterculus* is increased after the exposure to these compounds [217].

Considering that males are more sensitive to EOs, these compounds can also be exploited in male annihilation programs. The Male Annihilation Technique (MAT) involves the use of a high density of dispensers [218] or traps [219,220] triggered with a bait effective only towards males, combined with an insecticide, to reduce the male population of fruit flies [221]. ME and CL were used as attractants in MAT programs to eradicate *B. dorsalis* in Mariana Island and *B. tryoni* from Rapa Nui (Easter Island), respectively [222,223]. In IPM programs, MAT and SIT can be combined [36] as MAT can be performed before the release of the sterile males to reduce the population of wild males and to enhance the chance of SIT success.

However, though the premises are interesting, the use of EOs as lures in attract-and-kill programs in the field is still poorly investigated and implemented. For instance, Canale et al. [5] reported that the *R. officinalis*, *L. angustifolia*, and *H. suaveolens* EOs against *B. oleae* adults in semi-field conditions exerted a lower toxic effect for laboratory results at the same concentration. Thus, the authors hypothesized that the environmental conditions of semi-field experiments (e.g., temperature, light, in particular UV radiations), as well as the interaction between plant and EOs, could determine a decrease in their effectiveness because of their feeble stability in the environment [224]. Although the use of EOs in IPM programs is promising, there are still many doubts about their applicability in the field on a large scale. As reported by Pavela and Benelli [20], there are at least three reasons why EOs are not currently present on the market. Firstly, there is a lack of a standard procedure for the cultivation of plants and extraction of EOs from plant materials. The chemical composition of EOs, as well as their biological activity, varies depending on climatic and soil conditions. When the pedo-climatic conditions change, the concentration and presence of secondary metabolites in the plant may fluctuate [20]. A second concern regards the physio-chemical characteristics of EOs. EOs often display poor water solubility, scarce stability, high volatility, thermal decomposition, and oxidative degradation, which make them difficult to handle in field conditions [225]. In this regard, nanotechnology can help by encapsulating the EOs into nano or microemulsions, improving their physical and chemical stability, preventing the degradation of active agents, and enhancing the bioavailability of EOs [225,226,227]. A final constraint is the authorization process behind the commercialization of the EOs as bio-pesticides. In the European context, EOs may be considered as “basic substances (BSs)”, a new term introduced by the European Pesticides Regulation (EC) No. 1107/2009. However, EOs may fall under this definition only if they do not undergo any further formulation changes, such as the addition of emulsifiers, which makes the approval process for EOs much more difficult. Furthermore, several members of the European Commission are worried about the potential mutagenic or genotoxic adverse effects of EOs on the human endocrine system, based on a lack of relevant toxicological data on this topic. However, this seems an unfounded fear, considering that most of the negative effects of EOs appear at high dosages, with the application of undiluted EOs, or after long-term exposure. From a toxicological point of view, the toxicity of EOs towards mammals (LC_50_) is >1000 mg kg^−1^, except for some EOs such as boldo (*Peumus boldus* Molina, Monimiaceae), red cedar (*Juniperus virginiana* L., Cupressaceae), and pennyroyal (*M. pulegium*) whose toxicity is 130, 830 and 400 mg kg^−1^, respectively [228]. Moreover, due to the fumigation techniques and degradation properties of EOs, residues on plants are likely minimal [40]. Given that most of the EOs cannot be considered high-risk substances for human health.

Nonetheless, further evaluations on the efficacy, plant safety, and social and environmental impact of EOs are needed, and prospects for the application should be clarified [229]. The use of EOs for sustainable agricultural practices seems promising, and extensive research will probably clarify or deny their relevance in diverse applications. Due to their intrinsic characteristics, the pest control properties of EOs are usually very transitory and less effective than synthetic products. However, EOs can be an efficient alternative to conventional plant protection products when properly formulated and integrated with other sustainable pest management strategies.

## 5. Conclusions, Future Perspectives, and Challenges

Although the impressive increase in the number of publications involving botanical insecticides observed in the last years, the use of EOs as control tools against tephritid flies still represents a niche sector requiring further research. The increasing interest in EOs derives from their wide availability in nature, relatively low cost, and the belief that plant-borne extracts are non-toxic to humans and environmentally friendly. EOs may act as an attractant, increasing the mating success of males, or as toxics, showing noticeable acute toxicity toward the target insects. The same EO or the same compound can elicit both, as in the above-discussed case of limonene [33,46]. EOs properties can be applied in SIT and MAT programs to increase their success, or in lure and kill programs once all the limits related to their physicochemical properties are overcome. However, to extend the use of EOs to the field, it is necessary to standardize the methodologies behind their development, considering the phenological stage and/or the plant part from which they are extracted, and the pedoclimatic conditions of plant growing areas that can affect the relative number of bioactive compounds in the EOs. At the same time, further trials are needed to ascertain the biocidal or attractive activity of EOs and their main components on a larger number of pest species, as well as to validate the effectiveness of EOs obtained in different years and different geographical regions. In addition, studies to estimate the potential impact of EOs on the environment (i.e., non-target species) and human health should be implemented. Therefore, a multidisciplinary approach is strongly recommended to overcome EOs limits, to guarantee their efficacy and safety, and to create EO-based insecticide formulations to be adopted in IPM programs to control tephritids as well as other insects of agricultural and veterinary interest.

Box 1 **Essential oils as biopesticides: Advantages and constraints.**

Overall, the use of EOs as botanical pesticides has numerous advantages:

- Multiple mechanisms of action, therefore the development of resistance is unlikely.
- Low toxicity towards non-target organisms (including humans).
- Low health risk throughout the application due to their limited toxicity.
- High effectiveness towards a wide range of pests of agricultural, veterinary, and medical interest.

EOs have some constraints that represent key challenges for future research:

- Strict legislation.
- Uneven EO chemical composition depending on cultivation, harvesting and extraction conditions.
- Phytotoxic properties to crops and other non-target plant species.
- EO physio-chemical properties, such as thermolability and washability, reduce their stability and efficacy in field conditions.

Box 2 **Mating systems of tephritid fruit flies.**

The mating system of Tephritidae varies from species to species. Most tephritid flies are lekking species (i.e., leks are aggregations of males formed solely for mating) and they do not rely on resource-based mating systems [230]. Tephritid lekking sites are focal places in which male-male competition for partners (intra-sexual selection) and active choice of males by females (inter-sexual selection) drive the evolution of sexual traits [231,232]. Generally, lekking males initiate sexual behavior by releasing long-range pheromones that attract females to behavioral exhibition sites [233,234]. Then, females discriminate among lek participants and copulate with males that perform the best courtship behavior sequence, which includes wing movements combined with olfactory and tactile cues, before mounting attempts [8,232]. Females encounter several potential partners at the lek and are free (i.e., not coerced) to select their mate. This results in female marked choosiness [8], see also [235]. In many tephritid species, (e.g., the medfly, *C. capitata*, and the olive fruit fly, *B*. *oleae*), the courtship is preceded by male-male aggressive interactions for courtship sites, which are highly ritualized, and composed of several displays, including wing waving, pouncing, wing strikes, and boxing [236,237].

## Data Availability

Not applicable.
